# A 90-day safety study of meat from MSTN gene-edited Mongolian cattle in mice

**DOI:** 10.1038/s41598-025-31934-x

**Published:** 2026-01-15

**Authors:** Jia-Hao Chen, Hong-Yu Gong, Zhao-Yu Wen, De-Zheng Wang, Tao Wang, Li-Shuang Song, Xue-Fei Liu, Guang-Peng Li, Chun-Ling Bai, Lei Yang

**Affiliations:** State Key Laboratory of Reproductive Regulation and Breeding of Grassland Livestock, Inner Mongolia University, Hohhot, 010040 China

**Keywords:** Biochemistry, Physiology, Zoology

## Abstract

The knockout (KO) of the myostatin (MSTN) gene can increase muscle production in Mongolian cattle; however, the safety of MSTN-KO beef has not been evaluated. In this study, we fed mice varying concentrations of MSTN-KO beef and monitored physiological and tissue changes. Compared with the control group fed with wild-type beef, mice fed with MSTN-KO beef did not show significant changes, including weight gain, food intake, and organ weight. Furthermore, most blood parameters of the experimental groups remained stable. Serum metabolomics analysis confirmed that MSTN-KO beef had a limited impact on the mice’s overall metabolism, with only 24 differential metabolites identified. Our findings from this 90-day trial show no toxic effects of MSTN-KO Mongolian beef on mice. This directly addresses the long-standing lack of toxicity data for such gene-edited beef. Notably, this is the first study to fill the research gap, and the evidence generated in this work actively supports the safety assessment of MSTN-modified animal-derived foods.

**Supplementary Information:**

The online version contains supplementary material available at 10.1038/s41598-025-31934-x.

## Introduction

Mongolian cattle are one of the excellent local breeds from northern China, with numerous outstanding characteristics, including tender meat quality, remarkable resistance to adversity, strong reproductive capabilities, and ease of breeding and management. These advantages have been confirmed in related studies^1^. Mongolian cattle can adapt well to harsh ecological conditions such as cold, semi-desert grasslands, and desert grazing areas^2^, and they also have the potential to produce high-quality beef. However, compared to natural “double-muscled” breeds like Belgian Blues^3^, as well as other breeds like Angus and Simmental, Mongolian cattle have certain limitations, such as longer growth cycles, relatively lower meat production efficiency, and lower conversion rates. To address these limitations, our research centered on the MSTN gene. Leveraging gene-editing technology, we suppressed its expression in Mongolian cattle, resulting in the development of a new cattle strain. This novel variety not only substantially boosts growth rate and meat production, but also drastically shortens the breeding and improvement cycle, all while preserving the full suite of desirable traits inherent to the original Mongolian cattle. To summarize the method applied in this study, we focused on cultivating high-yield, “double-muscled” high-quality beef cattle using gene-editing technology — a type of modern biotechnology.

Myostatin (MSTN) is a member of the transforming growth factor β(TGF-β) superfamily. MSTN is primarily expressed in skeletal muscle and plays a negative regulatory role in muscle growth and development, with tissue-specific expression in muscle cells^4^. After being synthesized inside the cell, myostatin is transported and secreted into the extracellular space, where it circulates through the blood to regulate other tissues and cells^5,6^. The MSTN gene shows a high degree of evolutionary conservation across different species, indicating its crucial regulatory role in animal growth, development, and body patterning. CRISPR/Cas9 technology was employed to introduce MSTN point mutations. In recent years, advances in high-throughput sequencing, proteomics, and phosphoproteomics have significantly clarified research into the complex regulatory mechanisms of MSTN. Specifically, this includes MSTN’s roles in muscle growth, metabolism, and the pathogenesis of related diseases. Numerous studies have shown that MSTN is not only directly associated with skeletal muscle growth and development, but also involved in physiological processes such as glucose metabolism, lipid metabolism, and protein metabolism^7^. In addition, the MSTN gene knockout mouse model has demonstrated significant resistance to white fat accumulation and lipid metabolism disorders induced by T2DM (Type 2 Diabetes Mellitus), revealing that the MSTN gene may also be closely related to the onset and progression of metabolic diseases such as diabetes and obesity. This provides new insights for further investigation into the mechanisms of diabetes and obesity, as well as for clinical interventions and treatments^8–10^.

Currently, many countries, such as the United States, Canada, and Australia, classify gene-edited crops that do not involve the introduction of foreign genes as non-GMO organisms and have adopted relatively lenient management policies for these crops^11^. The U.S. Department of Agriculture has explicitly stated that it will not impose regulatory measures on crops developed using new breeding technologies. Meanwhile, the U.S. Food and Drug Administration (FDA) also believes that gene-edited foods that do not contain foreign genes are identical in composition to naturally grown foods, and therefore do not need to be specially labeled for differentiation. The safety of gene-edited plants and animals cannot be generalized; it must be judged specifically based on whether the edited gene and its natural mutation are substantially equivalent^12^. If these natural mutations have been considered safe after long-term consumption, then traits changed through gene editing to achieve the same effect as natural mutations can also be considered safe. The team led by Ma Li-Jia at West Lake University has modified the PE system to improve in vivo off-target analysis and chromosomal translocation identification related to gene editing, providing a new approach for gene editing safety assessment^13^. In China, the Ministry of Agriculture and Rural Affairs released the 2024 list of approved biosafety certificates for genetically modified agricultural organisms, which also reflects the country’s recognition of gene editing technology in food production.

Under the same feeding volume and management conditions, gene-edited animals exhibit significant improvements in meat yield and enhanced disease resistance, among many other advantages^14^. This not only increases resource utilization but also effectively reduces the environmental burden caused by livestock farming. To date, numerous MSTN-mutant animals have been successfully bred, such as the MSTN knockout Meishan pigs developed by the Institute of Animal Science, Chinese Academy of Agricultural Sciences (IAS) in 2015^15^. However, despite the policy support and technical progress, there remains a critical gap: current safety evaluations for gene-edited foods are insufficient, especially for MSTN gene-edited Mongolian beef—no research reports have yet assessed its safety in mammals. While the economic value of gene-edited animals is a focus, public concerns about potential hidden risks of food derived from them persist, and global regulatory policies for gene-edited foods still vary^16^. In light of this, to promote the commercialization of gene-edited large livestock and ensure the safety of such products for human consumption, a comprehensive and rigorous biosafety evaluation must be conducted when developing new genome editing-related products^17^, to ensure that consuming these gene-edited animals will not cause any adverse health effects on consumers. Referencing the European Food Safety Authority’s recommendation for a 90-day oral toxicity study in rodents^18^, as well as the “National Food Safety Standard 90-Day Oral Toxicity Test”(GB 15193.13–2015), we conducted a 90-day subacute toxicity test on KM mice to assess the safety of MSTN gene-edited Mongolian cattle in mammals^19^.

## Results

### Physiology and clinical observation

All animals survived during the 90-day feeding period, with no deaths. The skin and fur of the mice in each group were normal, showing no hair loss or necrosis. The mice’s movement was normal, with no hyperactivity, hypersensitivity, paralysis, or prone posture. No secretion was observed in the eyes, and no nasal discharge was seen. The reactions of all groups were normal (Table 1).

**Table 1:** Hematological parameters of males in the studied groups on the 90th day. Each value is presented as mean ± SD. WBC: white blood cell Count; LYM#: lymphocyte Count; LYM%: lymphocyte Percentage; MON#: monocyte Count; MON%: monocyte Percentage; EOS#: eosinophil Count; EOS%: eosinophil Percentage; BAS#: basophil Count; BAS%: basophil Percentage; NEU#: neutrophil Count; NEU%: neutrophil Percentage; RBC: red blood cell Count; HCT: Hematocrit; MCV: mean corpuscular Volume; RDW: red cell distribution Width; HGB: hemoglobin Concentration; MCH: mean corpuscular hemoglobin; MCHC: mean corpuscular hemoglobin Concentration; PLT: platelet Count; PCT: plateletcrit. PDW: platelet distribution Width; MPV: mean platelet Volume. * *p*<0.05、 ** *p*<0.01、*** *p*<0.001 compared with the Con group.

	Con	MG-WT10%	MG-WT20%	MG-WT30%	MG-MT10%	MG-MT20%	MG-MT30%
WBC	5.66 ± 0.65	5.68 ± 0.59	5.48 ± 0.45	5.32 ± 0.18	5.74 ± 0.61	5.4 ± 0.29	5.96 ± 0.18
LYM#	6.36 ± 1.07	5.58 ± 1.27	5.80 ± 0.80	6.24 ± 0.61	6.12 ± 1.09	5.22 ± 0.52 *	4.90 ± 0.58 *
LYM%	73.50 ± 3.32	71.12 ± 2.76	70.94 ± 3.06	72.34 ± 2.13	67.78 ± 4.89*	68.9 ± 3.15	70.64 ± 2.29
MON#	0.66 ± 0.23	1.02 ± 0.15 *	0.62 ± 0.37	0.86 ± 0.23	0.70 ± 0.16	0.78 ± 0.24	0.56 ± 0.13
MON%	8.62 ± 2.79	8.18 ± 3.10	10.58 ± 2.46	9.32 ± 3.01	7.82 ± 2.37	9.24 ± 2.69	9.16 ± 2.78
EOS#	0.25 ± 0.07	0.23 ± 0.04	0.15 ± 0.03 *	0.26 ± 0.1	0.21 ± 0.06	0.12 ± 0.05 **	0.22 ± 0.08
EOS%	2.80 ± 0.85	2.28 ± 0.61	2.02 ± 0.56	3.3 ± 0.63	2.20 ± 0.84	2.52 ± 0.36	3.28 ± 0.75
BAS#	0.07 ± 0.02	0.06 ± 0.02	0.03 ± 0.02	0.06 ± 0.03	0.06 ± 0.03	0.05 ± 0.03	0.06 ± 0.03
BAS%	0.57 ± 0.16	0.40 ± 0.20	0.51 ± 0.27	0.33 ± 0.2	0.44 ± 0.26	0.44 ± 0.13	0.52 ± 0.1
NEU#	1.94 ± 0.63	1.60 ± 0.64	1.72 ± 0.73	1.4 ± 0.8	1.66 ± 1.05	1.78 ± 0.59	2.3 ± 0.97
NEU%	26.8 ± 7.55	31.38 ± 7.61	29.96 ± 6.73	23.22 ± 5.59	28.34 ± 5.3	26.46 ± 7.86	27.46 ± 3.73
RBC	4.80 ± 0.41	5.00 ± 0.46	4.20 ± 0.76 *	4.44 ± 0.55	4.42 ± 0.38	4.06 ± 0.55 *	4.46 ± 0.19
HCT	43.6 ± 2.49	43.72 ± 4.61	43.64 ± 4.73	43.64 ± 4.73	45.4 ± 4.54	44 ± 3.6	43.24 ± 3.02
MCV	46.12 ± 1.72	47.18 ± 2.56	46.94 ± 2.31	47.16 ± 2.94	47.68 ± 2.42	47.26 ± 2.42	49.26 ± 2.31
RDW	12.26 ± 2.53	12.14 ± 2.59	13.44 ± 1.39	11.38 ± 2.92	13.16 ± 0.85	13.36 ± 2.58	11.04 ± 2.21
HGB	140.60 ± 21.73	128.80 ± 9.36	140.40 ± 29.88	129.00 ± 15.12	136.00 ± 14.58	148.40 ± 12.14	136.60 ± 26.58
MCH	14.94 ± 0.90	14.50 ± 0.60	15.8 ± 1.20	15.52 ± 1.23	14.98 ± 0.79	16.06 ± 0.91	15.32 ± 1.08
MCHC	35.64 ± 0.78	35.64 ± 0.81	35.3 ± 1.08	35.5 ± 0.81	35.62 ± 0.96	35.14 ± 0.43	34.56 ± 0.53
PLT	508.4 ± 141.02	586 ± 126.55	556.2 ± 76.07	565.2 ± 91.05	655.6 ± 95.05	503.4 ± 140.4	576 ± 50.92
PCT	0.29 ± 0.11	0.31 ± 0.10	0.34 ± 0.15	0.37 ± 0.12	0.33 ± 0.15	0.36 ± 0.10	0.30 ± 0.15
PDW	23.38 ± 3.00	23.46 ± 1.88	22.54 ± 3.61	21.86 ± 2.13	23.6 ± 3.03	23.42 ± 3.03	23.94 ± 3.76
MPV	12.26 ± 2.41	10.94 ± 2.92	10.86 ± 2.39	10.02 ± 1.90	10.7 ± 2.76	11.24 ± 3.93	12.16 ± 3.38

### Body weight

**Fig. 1:**
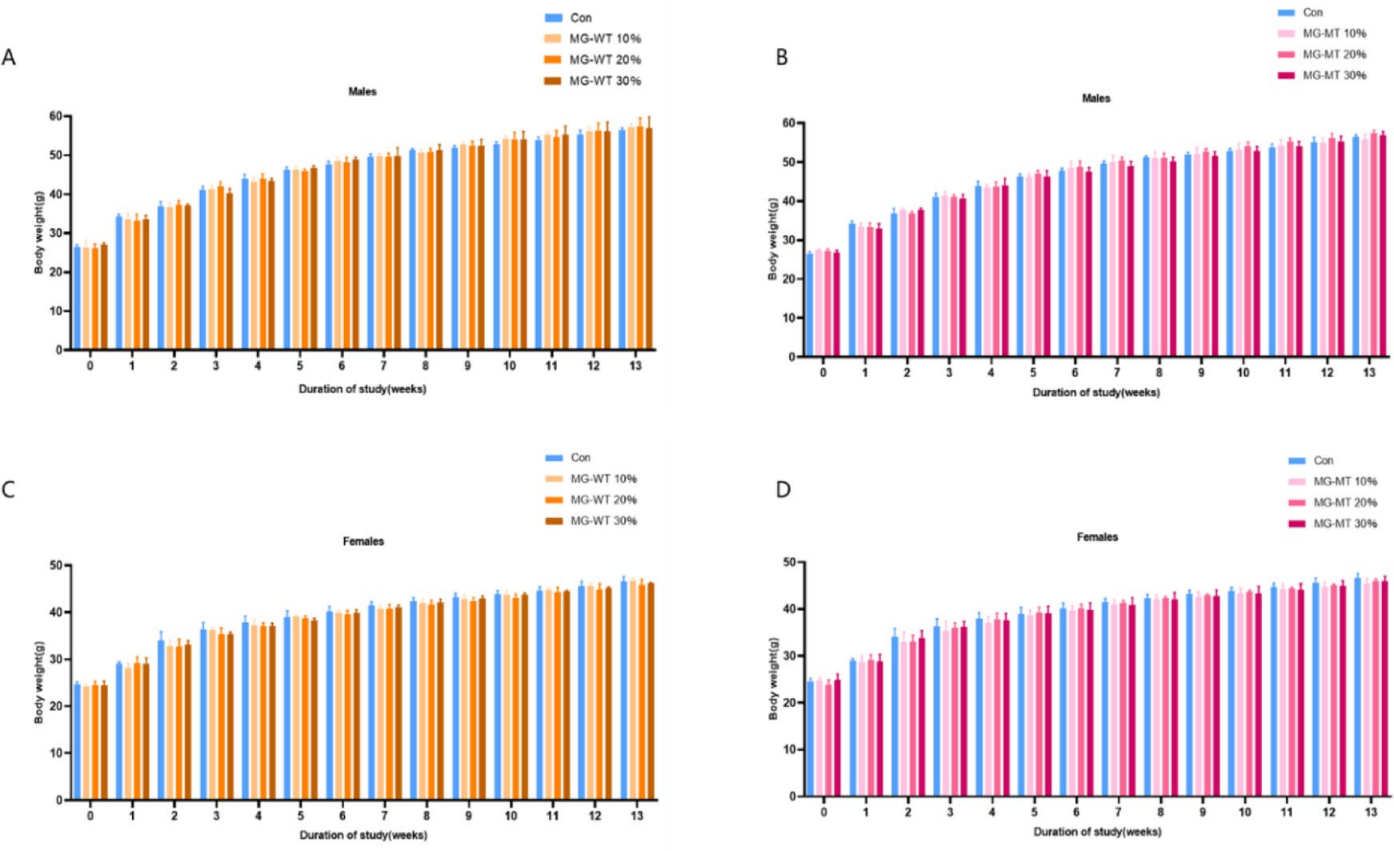
Weekly Mean Body Weight of Male and Female Mice Fed Control Diet or Different Doses of MG-WT/MG-MT Diets Over the Experimental Period. (**A**) Male mice fed control (Con) diet or MG-WT diets (10%, 20%, 30%); (**B**) Male mice fed control (Con) diet or MG-MT diets (10%, 20%, 30%); (**C**) Female mice fed control (Con) diet or MG-WT diets (10%, 20%, 30%); (**D**) Female mice fed control (Con) diet or MG-MT diets (10%, 20%, 30%).

The body weight of mice in each group gradually increased with the extension of feeding time, with no abnormalities observed (Fig. 1). There were no significant differences in the initial body weight, final body weight, and average daily weight gain between the experimental groups and the control group (*P* > 0.05), indicating that the addition of different levels of MSTN gene-edited Mongolian beef did not have any adverse effects on weight gain or the growth performance of the mice.

### Mean weekly feed utilization

**Fig. 2:**
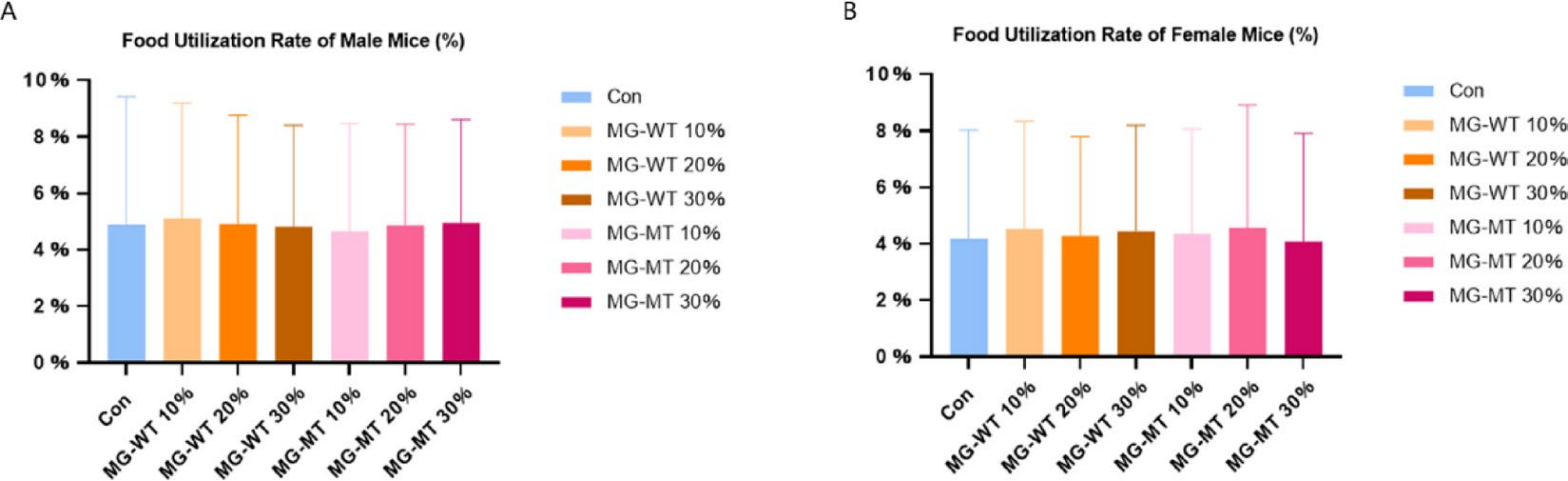
Mean Food Utilization Rate (%) of Male and Female Mice After 90 Days of Feeding With Different Diets (Control, MG-WT30%, MG-MT30%). (**A**) Male mice; (**B**) Female mice.

The amount of food consumed is calculated as the difference between the amount of feed given at the start of the experimental period and the remaining feed amount after one week. The weekly food intake data for each cage of mice are recorded. Throughout the study, there were no statistically significant differences in the average weekly feed consumption among the groups, and no significant differences in the average weekly food utilization rate (%) between groups (*P* > 0.05) (Fig. 2).

### Hematology

Male mouse white blood cell indicators: The lymphocyte count in the commercial feed control group showed statistical differences, with the MSTN gene-edited beef MG-MT 20% group and the MSTN gene-edited beef MG-MT 30% group decreasing (*P* = 0.043, *P* = 0.01) compared to the control group; The lymphocyte percentage in the commercial feed control group showed statistical differences, with the MSTN gene-edited beef MG-MT 10% group decreasing (*P* = 0.023) compared to the control group; The eosinophil count in the commercial feed control group showed a statistically significant difference, with the MSTN gene-edited beef MG-MT 20% group and the wild Mongolian beef MG-WT 20% group decreasing (*P* = 0.009, *P* = 0.043) compared to the control group; The monocyte count in the commercial feed control group showed statistical differences, with the wild Mongolian beef MG-WT 10% group increasing (*P* = 0.019) compared to the control group. Male mouse red blood cell indices: The red blood cell count in the commercial feed control group showed a statistical difference, with the MSTN gene-edited beef MG-MT 20% group and the wild Mongolian beef MG-WT 20% group decreasing (*P* = 0.016, *P* = 0.048) compared to the control group (Table 1). Female mouse white blood cell indices: The percentage of lymphocytes in the commercial feed control group showed a statistical difference, with the wild Mongolian beef MG-MT 10% group decreasing (*P* = 0.047, *P* = 0.017) compared to the control group; the eosinophil count in the commercial feed control group showed a highly significant statistical difference, with the MSTN gene-edited beef MG-MT 30% group increasing (*P* < 0.001) compared to the control group. Additionally, there were no significant differences in other blood indices between the groups (*P* > 0.05) (Table 2). Statistical data showed that some mice had lower values in their hematological indices, which led to a considerable decrease in the average value and thus a difference from the control group, but the indices for these mice were still within the normal range. Based on the above test results, although there are statistical differences in certain individual indicators, the blood routine data of mice in all groups are within the normal range, and no abnormal changes associated with feeding MSTN gene-edited beef were observed. Furthermore, these differences are unrelated to the dosage and are likely due to individual variations in the mice, rather than being associated with feeding MSTN gene-edited Mongolian beef.

**Table 2:** Hematological parameters of females in the studied groups on the 90th day. Each value is presented as mean ± SD. WBC: white blood cell Count; LYM#: lymphocyte Count; LYM%: lymphocyte Percentage; MON#: monocyte Count; MON%: monocyte Percentage; EOS#: eosinophil Count; EOS%: eosinophil Percentage; BAS#: basophil Count; BAS%: basophil Percentage; NEU#: neutrophil Count; NEU%: neutrophil Percentage; RBC: red blood cell Count; HCT: Hematocrit; MCV: mean corpuscular Volume; RDW: red cell distribution Width; HGB: hemoglobin Concentration; MCH: mean corpuscular hemoglobin; MCHC: mean corpuscular hemoglobin Concentration; PLT: platelet Count; PCT: plateletcrit. PDW: platelet distribution Width; MPV: mean platelet Volume. * *p*<0.05、 ** *p*<0.01、*** *p*<0.001 compared with the Con group.

	Con	MG-WT10%	MG-WT20%	MG-WT30%	MG-MT10%	MG-MT20%	MG-MT30%
WBC	5.76 ± 0.56	6.08 ± 0.40	6.10 ± 0.30	5.56 ± 0.39	5.88 ± 0.45	6.18 ± 0.30	5.74 ± 0.36
LYM#	4.92 ± 1.05	5.86 ± 0.52	4.74 ± 0.70	4.90 ± 1.02	4.66 ± 0.81	4.76 ± 0.82	5.36 ± 0.89
LYM%	60.58 ± 3.03	65.32 ± 3.40 *	62.24 ± 4.41	62.6 ± 2.07	62.9 ± 2.06	60.06 ± 1.78	62.48 ± 1.55
MON#	0.62 ± 0.16	0.72 ± 0.22	0.66 ± 0.17	0.84 ± 0.34	0.80 ± 0.31	0.86 ± 0.21	0.74 ± 0.24
MON%	8.72 ± 2.44	8.16 ± 2.85	9.66 ± 2.76	8.10 ± 3.36	8.12 ± 1.02	10.04 ± 1.81	9.14 ± 1.48
EOS#	0.21 ± 0.13	0.32 ± 0.04 *	0.26 ± 0.07	0.27 ± 0.04	0.29 ± 0.03	0.25 ± 0.06	0.37 ± 0.11 ***
EOS%	2.22 ± 0.08	2.22 ± 0.35	2.10 ± 0.62	2.20 ± 0.50	2.10 ± 0.25	2.12 ± 0.48	2.26 ± 0.40
BAS#	0.08 ± 0.02	0.05 ± 0.02	0.05 ± 0.04	0.03 ± 0.02	0.05 ± 0.03	0.05 ± 0.03	0.06 ± 0.03
BAS%	0.53 ± 0.14	0.58 ± 0.21	0.41 ± 0.24	0.42 ± 0.20	0.29 ± 0.15	0.41 ± 0.26	0.47 ± 0.30
NEU#	1.92 ± 0.99	1.84 ± 0.70	2.52 ± 0.86	1.88 ± 0.97	1.38 ± 0.22	2.30 ± 0.60	1.68 ± 0.61
NEU%	28.9 ± 7.13	25.16 ± 7.8	27.16 ± 5.81	26.7 ± 5.41	26.46 ± 6.22	24.76 ± 5.19	22.94 ± 3.95
RBC	4.78 ± 0.75	4.62 ± 0.55	4.28 ± 0.43	4.68 ± 0.64	4.70 ± 0.67	5.06 ± 0.29	4.48 ± 0.54
HCT	42.9 ± 2.83	42.62 ± 2.05	47.16 ± 2.53	45.2 ± 2.21	44.00 ± 4.26	43.86 ± 3.40	42.30 ± 3.70
MCV	49.1 ± 2.29	48.24 ± 2.48	47.64 ± 2.30	47.6 ± 3.25	47.88 ± 2.37	48.6 ± 3.18	50.04 ± 2.52
RDW	12.44 ± 1.68	9.96 ± 1.18	12.88 ± 1.82	12.16 ± 1.20	13.78 ± 1.54	11.34 ± 2.29	10.70 ± 1.38
HGB	153.4 ± 7.37	146.8 ± 23.75	135.6 ± 20.95	149.2 ± 25.14	128.8 ± 19.15	140.00 ± 21.64	131.00 ± 8.49
MCH	14.72 ± 1.28	15.14 ± 1.20	15.14 ± 1.11	15.24 ± 1.28	14.70 ± 0.82	14.46 ± 0.65	15.44 ± 0.75
MCHC	35.26 ± 1.02	35.84 ± 1.05	35.50 ± 0.65	35.64 ± 0.96	35.12 ± 1.22	35.7 ± 0.62	35.94 ± 0.54
PLT	542.4 ± 73.2	634.4 ± 20.95	546.8 ± 55.56	536.2 ± 71.89	583.6 ± 66.4	519 ± 90.88	525.6 ± 73.36
PCT	0.30 ± 0.15	0.28 ± 0.11	0.28 ± 0.13	0.26 ± 0.10	0.38 ± 0.16	0.40 ± 0.15	0.39 ± 0.16
PDW	24.78 ± 1.62	23.84 ± 2.70	24.14 ± 2.28	24.76 ± 1.93	25.34 ± 1.72	24.96 ± 1.71	25.66 ± 1.52
MPV	10.82 ± 1.08	11.38 ± 1.14	10.88 ± 3.08	10.76 ± 3.04	11.04 ± 1.07	10.40 ± 1.71	11.56 ± 2.01

**Table 3:** Relative organ weight/body weight of tissues in the male KM mice. Each value is presented as mean ± SD. * *p*<0.05、** *p*<0.01、*** *p*<0.001 compared with the Con group.

	Con	MG-WT10%	MG-WT20%	MG-WT30%	MG-MT10%	MG-MT20%	MG-MT30%
Heart	0.459% ± 0.024%	0.448% ± 0.030%	0.419% ± 0.030%	0.453% ± 0.030%	0.448% ± 0.030% *	0.419% ± 0.030%	0.453% ± 0.030%
Liver	5.361% ± 0.186%	5.411% ± 0.178%	5.232% ± 0.339%	5.468% ± 0.365%	5.411% ± 0.178%	5.232% ± 0.339%	5.468% ± 0.365%
Spleen	0.339% ± 0.025%	0.325% ± 0.048%	0.315% ± 0.037%	0.307% ± 0.033%	0.325% ± 0.048%	0.315% ± 0.037%	0.307% ± 0.033%
Lung	0.488% ± 0.023%	0.485% ± 0.023%	0.489% ± 0.018%	0.471% ± 0.023%	0.485% ± 0.023%	0.489% ± 0.018%	0.471% ± 0.023%
Left kidney	0.748% ± 0.025%	0.765% ± 0.043%	0.715% ± 0.039%	0.718% ± 0.060%	0.765% ± 0.043%	0.715% ± 0.039%	0.718% ± 0.060%
Right kidney	0.771% ± 0.019%	0.758% ± 0.021%	0.726% ± 0.041%	0.753% ± 0.064%	0.758% ± 0.021%	0.726% ± 0.041%	0.753% ± 0.064%
Testis	0.540% ± 0.021%	0.525% ± 0.042%	0.476% ± 0.051%	0.520% ± 0.047%	0.525% ± 0.042%	0.476% ± 0.051%	0.520% ± 0.047%

## Non-targeted serum metabolomics: principal component analysis (PCA)

### PCA analysis

**Fig. 3:**
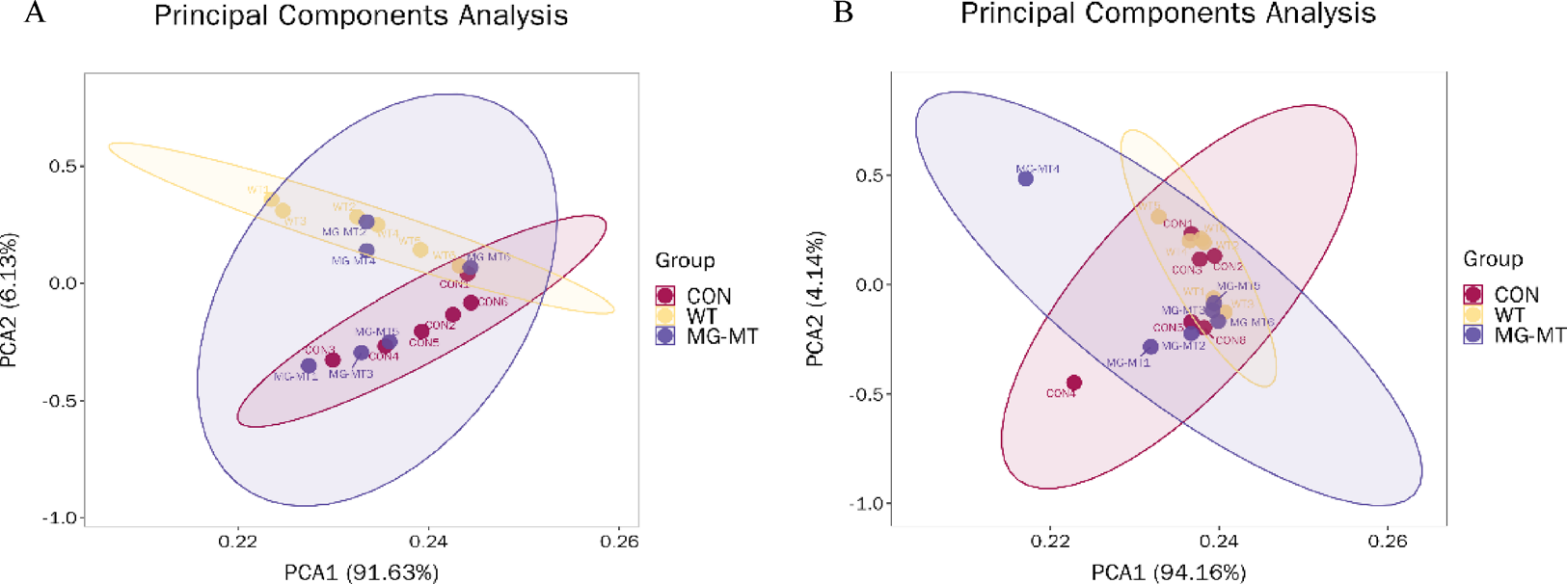
Principal Component Analysis (PCA) of Serum Metabolites in Male and Female Mice Fed Different Diets (*n* = 6). Points of different colors represent distinct experimental groups: CON (control group, red dots); MG-WT30% (30% wild-type Mongolian beef feeding group, yellow dots); MG-MT30% (30% MSTN gene-edited Mongolian beef feeding group, blue dots). The horizontal axis (PC1) and vertical axis (PC2) represent the scores of the first and second principal components, respectively. (**A**) Male mice; (**B**) Female mice.

In order to investigate the impact of adding MSTN gene-edited Mongolian beef feed on the serum metabolites of mice, and to determine whether there are significant differences and assess the system stability of the test, we performed principal component analysis (PCA) on the serum metabolomics data of male and female KM mice. The x-axis represents the first principal component score (PC1), while the y-axis represents the second principal component score (PC2), with each point representing a sample, and different groups displayed using different colors. The relative position of each point represents the degree of dispersion between samples, with a closer distance between points indicating more similar expression patterns. In male mice, the variance contribution rate of the first principal component (PC1) was 91.63%, the variance contribution rate of the second principal component (PC2) was 6.13%, and the cumulative variance contribution rate of the two components was 97.76% (Fig. 3A). In female mice, the variance contribution rate of the first principal component (PC1) was 94.16%, the variance contribution rate of the second principal component (PC2) was 4.14%, and the cumulative variance contribution rate of the two components was 98.3%(Fig. 3B). It can be seen that the six replicate serum samples from each group of mice are tightly clustered, with no significant separation, indicating that the differences within the group are small and the experimental reproducibility is good. Additionally, there is a significant interaction between the male and female mouse groups, suggesting that the differences in metabolite content between the groups are relatively small.

### Non-targeted serum metabolomics: screening and characterization of differential metabolites

**Fig. 4:**
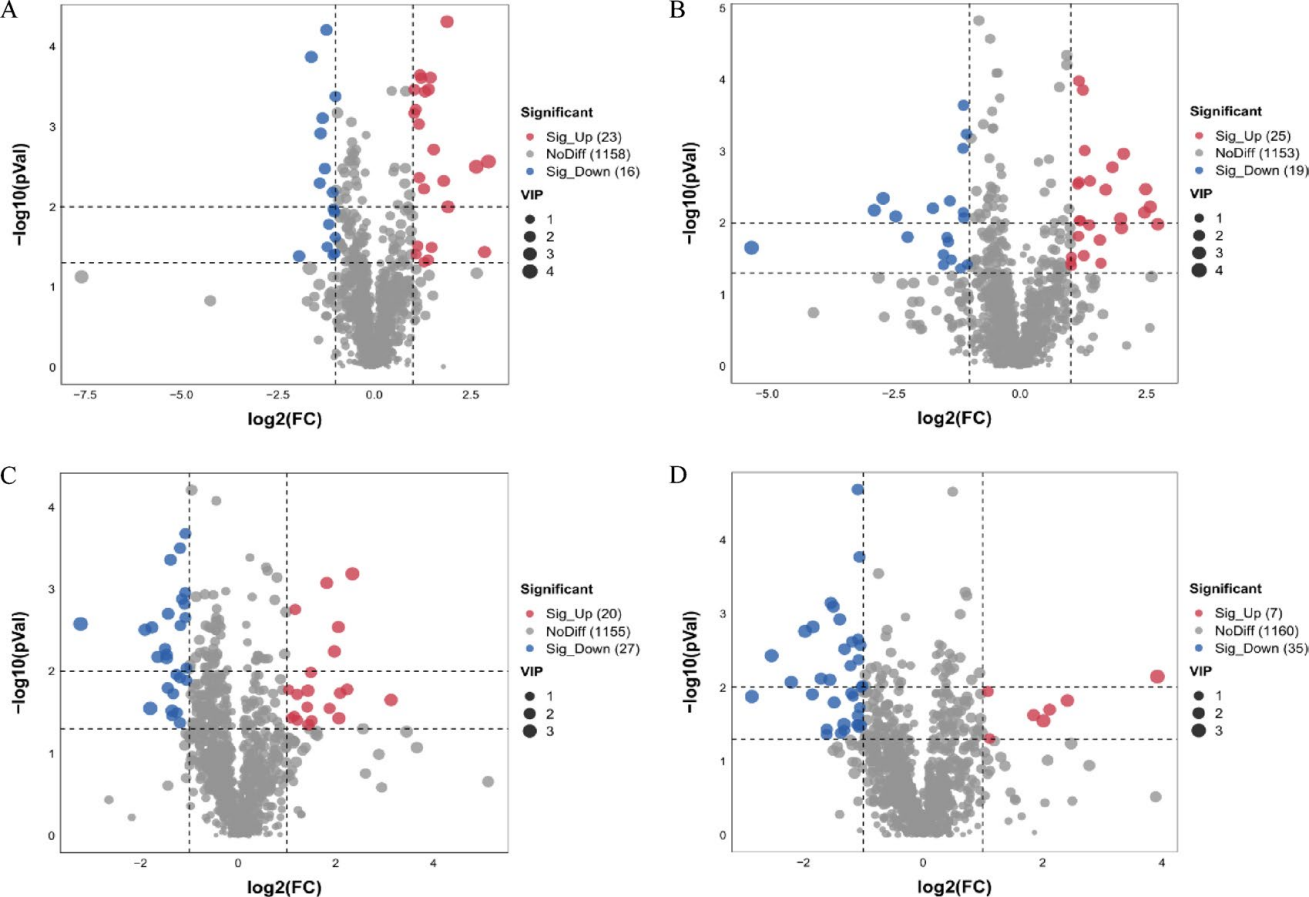
Volcano Plots of Serum Differential Metabolites in Male and Female Mice: Comparisons Between Control Group and MG-WT30%/MG-MT30% Groups. Red indicates upregulated significantly differentially expressed metabolites, blue indicates downregulated significantly differentially expressed metabolites, and gray dots represent non-significantly differentially expressed metabolites. Each dot in the plot represents a metabolite. (**A**) Shows differential metabolites comparing the control group to the MG-WT 30% in the male mouse group. (**B**) Shows differential metabolites comparing the control group to MG-MT30% in the male mouse group, with the control group and MG-WT 30% as the background. The circled solid dots represent metabolites that are. Common between the comparison of the control group with MG-MT30% and the comparison of the control group with MG-WT 30%. (**C**) Shows differential metabolites comparing the control group to MG-WT 30% in the female mouse group. (**D**) Shows differential metabolites comparing the control group to MG-MT30% in the female mouse group, with the control group and MG-WT 30% as the background. The circled solid dots represent metabolites that are common between the comparison of the control group with MG-MT30% and the comparison of the control group with MG-WT 30%.

In order to further investigate the differences in the types and quantities of metabolites between groups, the fold change (FC) values of metabolites between groups were used, and the metabolite abundance values were log2-transformed to normalize the data to a normal distribution. A T-test statistical analysis was performed. Additionally, the VIP (Variable Important for the Projection) values from multivariate statistics were used to screen for differentially expressed metabolites, which were selected based on the following criteria: FC ≥ 2 or FC ≤ 0.5, p value < 0.05, and VIP ≥ 1. A volcano plot was created with log2(FC) on the x-axis and log10(p-value) on the y-axis, displaying all differentially expressed metabolites. Metabolites with no significant changes were represented in gray. The volcano plot analysis results showed that, in male mice, a total of 39 significantly different metabolites were observed between the control group and the MG-WT 30% group, with 23 metabolites upregulated and 16 downregulated (Fig. 4A); 44 significantly different metabolites were observed between the control group and the MG-MT 30% group, with 25 upregulated and 19 downregulated metabolites (Fig. 4B); Among these significant differential metabolites, five metabolites are simultaneously present in both comparison groups.

Similarly, in female mice, a total of 47 metabolites showed significant differences between the control group and the MG-WT 30% group, with 20 upregulated and 27 downregulated (Fig. 4C); between the control group and the MG-MT 30% group, 42 metabolites showed significant differences, including 7 upregulated and 35 downregulated metabolites (Fig. 4D). Among these significantly different metabolites, 19 were present in both comparison groups. The volcano plot results show no significant difference in metabolite expression between the control group and the two experimental groups (MG-WT 30% and MG-MT 30%) and reveal a shared trend of changes in certain metabolites across different comparison groups.

### Organ weights

There was a statistically significant difference in the hearts of male mice fed with commercial feed compared to those fed with wild Mongolian beef MG-WT 10% (*P* = 0.048) (Table 3). In addition, there were no significant differences between groups for the coefficients of other organs (*P* > 0.05). For female mice, there were no significant differences in organ coefficients between groups (*P* > 0.05) (Table 4). Based on the overall results, although a statistical difference was observed in the hearts of male mice fed with wild Mongolian beef MG-WT 10%, the coefficient was within the normal range, and no structural changes were observed in the heart tissue in histological sections.

**Table 4:** Relative organ weight/body weight of tissues in the female KM mice. Each value is presented as mean ± SD. * *p*<0.05、** *p*<0.01、*** *p*<0.001 compared with the Con group.

	Con	MG-WT10%	MG-WT20%	MG-WT30%	MG-MT10%	MG-MT20%	MG-MT30%
Heart	0.413% ± 0.028%	0.434% ± 0.025%	0.419% ± 0.034%	0.416% ± 0.044%	0.418% ± 0.023%	0.415% ± 0.032%	0.426% ± 0.022%
Liver	4.638% ± 0.288%	4.891% ± 0.394%	4.696% ± 0.183%	4.758% ± 0.360%	4.481% ± 0.342%	4.763% ± 0.238%	4.754% ± 0.253%
Spleen	0.294% ± 0.019%	0.289% ± 0.022%	0.276% ± 0.024%	0.300% ± 0.039%	0.307% ± 0.022%	0.283% ± 0.033%	0.283% ± 0.038%
Lung	0.494% ± 0.020%	0.548% ± 0.029%	0.512% ± 0.050%	0.505% ± 0.021%	0.469% ± 0.025%	0.477% ± 0.030%	0.487% ± 0.026%
Left kidney	0.618% ± 0.063%	0.635% ± 0.029%	0.610% ± 0.033%	0.599% ± 0.021%	0.603% ± 0.021%	0.617% ± 0.023%	0.585% ± 0.044%
Right kidney	0.606% ± 0.047%	0.629% ± 0.019%	0.620% ± 0.053%	0.608% ± 0.035%	0.602% ± 0.027%	0.607% ± 0.031%	0.578% ± 0.042%
Ovary	0.085% ± 0.017%	0.084% ± 0.010%	0.076% ± 0.006%	0.081% ± 0.009%	0.076% ± 0.006%	0.081% ± 0.007%	0.080% ± 0.008%

**Table 5:** Study design—dose groups. The table presents the number of male and female animals in each group.

Group	Diet	Number of animals/group	
		Male	Female
Con	Commercially available diet	12	12
MG-WT10%	10% wild-type control beef	6	6
MG-WT20%	20% wild-type control beef	6	6
MG-WT30%	30% wild-type control beef	12	12
MG-MT10%	10% MSTN-knockout beef	6	6
MG-MT20%	20% MSTN-knockout beef	6	6
MG-MT30%	30% MSTN-knockout beef	12	12

Con: the standard commercial feed for mice was used as the control group; MG - WT10%: the standard commercial feed for mice with beef derived from wild Mongolian Cattle added to replace 10% of the protein; MG - WT20%: the standard commercial feed for mice with meat derived from wild Mongolian Cattle added to replace 20% of the protein; MG - WT30%: the standard commercial feed for mice with beef derived from wild Mongolian Cattle added to replace 30% of the protein; MG - MT10%: the standard commercial feed for mice with meat derived from MSTN-knockout Mongolian Cattle added to replace 10% of the protein; MG - MT20%: the standard commercial feed for mice with beef derived from MSTN-knockout Mongolian Cattle added to replace 20% of the protein; MG - MT30%: the standard commercial feed for mice with meat derived from MSTN-knockout Mongolian Cattle added to replace 30% of the protein.

### Gross necropsy and histopathology

**Fig. 5:**
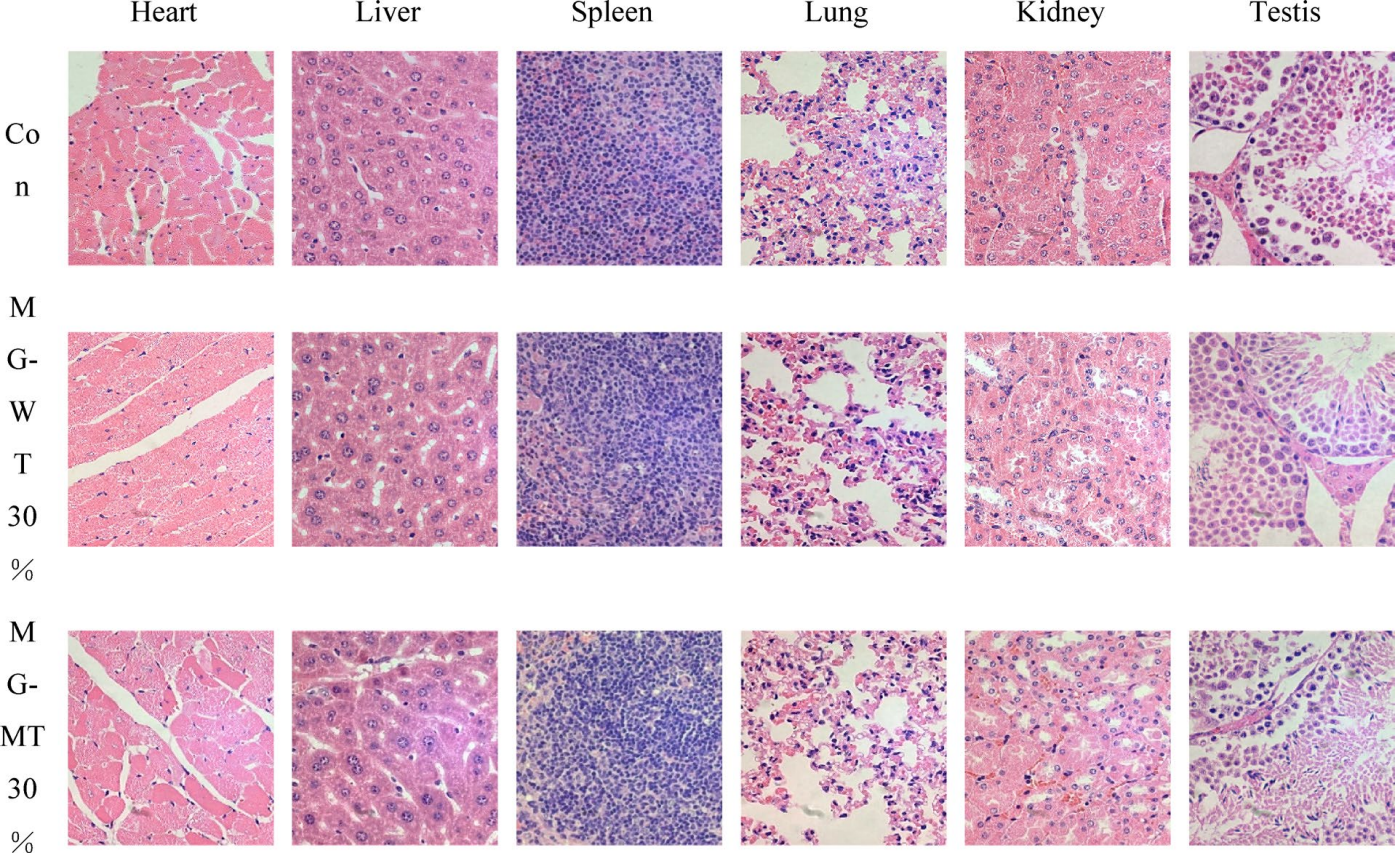
Histopathological Observations of Major Tissues in Mice After 90-Day Feeding (H&E Staining, 400X). Tissues examined include heart, liver, spleen, lung, kidney, and testis. Experimental groups are as follows: Con (Control group): Fed standard commercial mouse feed (consistent with the study’s control design); MG-WT30%: Fed standard commercial mouse feed, with 30% protein replaced by beef from wild-type Mongolian cattle; MG-MT30%: Fed standard commercial mouse feed, with 30% protein replaced by beef from MSTN gene-edited Mongolian cattle.

**Fig. 6:**
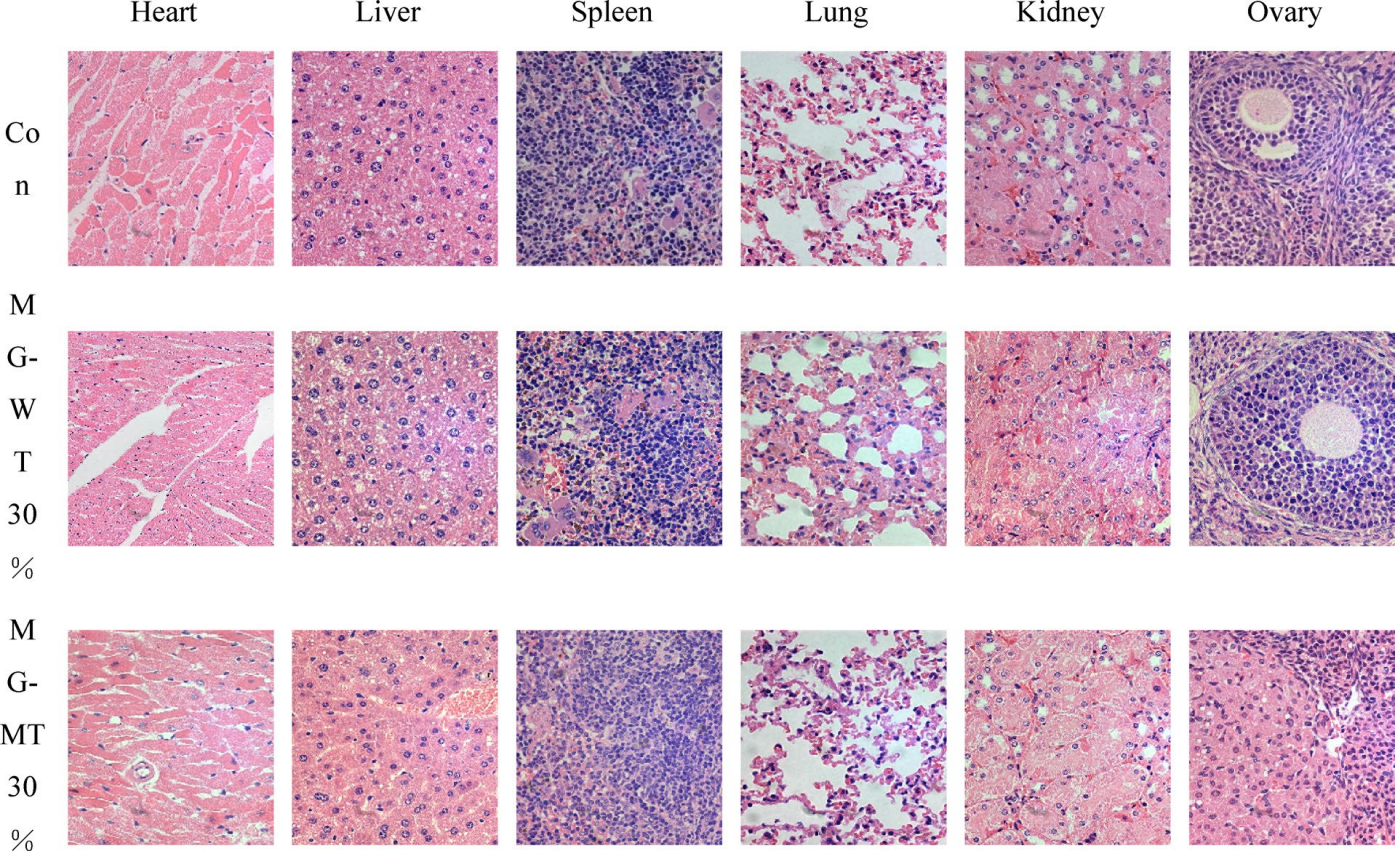
Histopathological Observations of Major Tissues in Mice After 90-Day Feeding (H&E Staining, 400X). Tissues examined include heart, liver, spleen, lung, kidney, and ovary. Experimental groups are as follows: Con (Control group): Fed standard commercial mouse feed (consistent with the study’s control design); MG-WT30%: Fed standard commercial mouse feed, with 30% protein replaced by beef from wild-type Mongolian cattle; MG-MT30%: Fed standard commercial mouse feed, with 30% protein replaced by beef from MSTN gene-edited Mongolian cattle.

Histopathological examination of HE-stained sections from all key organs under a microscope showed no obvious lesions in the brain, heart, lung, spleen, stomach, thymus, adrenal gland, skeletal muscle, male-specific testis, and female-specific ovary and uterus in selected male and female mice from all five feeding groups (Figs. 5 and 6).

## Discussion

In addition, the Ministry of Agriculture and Rural Affairs of China has released the list of approved biosafety certificates for genetically modified agricultural organisms in 2024, which also reflects China’s recognition of gene editing technology in the field of food production. With the advancement of science and technology, gene-edited food may gradually appear in people’s daily lives, making comprehensive safety assessments particularly important to ensure food safety for humans. However, there is currently a lack of safety evaluations for such foods, especially regarding the safety assessment of MSTN gene-edited Mongolian beef in mammals, with no relevant research reports available so far.

Our experimental results show that during the observation period of the subchronic toxicity study, no experimental animals exhibited signs of anorexia, diarrhea, or other toxic symptoms. The mice in all groups had smooth fur, good mental state, and their movement and response abilities were within normal levels, with no abnormal behaviors observed. Food intake and body weight changes are important indicators for assessing the health of mice, indirectly reflecting their growth, development, and metabolic levels, etc^20^. Compared with the data from the control group, no statistically significant differences were found in body weight, food intake, or feed utilization efficiency in the mice of the three experimental groups. This result indicates that the addition of the MSTN gene-edited Mongolian beef did not have any adverse effects on the mice’s growth performance, further confirming the safety of this gene-edited food at the subchronic toxicity level.

Hematological analysis provides us with detailed data on the overall health status of mice, serving as an important tool for assessing the potential toxic effects of any substance, accurately reflecting the physiological and pathological conditions of the mice^21^. The results of this hematological analysis reveal that the blood parameters of most experimental group animals are relatively stable, showing no significant changes. Notably, female mice in the MG-MT 30% group exhibited an extremely significant statistical increase (*P* < 0.001) in eosinophil count (EOS#) compared to the control group, with overall values being elevated. It is generally believed that elevated eosinophil counts (EOS#) may be associated with certain allergic reactions, skin inflammation, the occurrence of specific tumors, or rheumatic diseases^22–24^. However, during the daily clinical observations conducted twice a day in this experiment, no adverse symptoms such as skin inflammation were observed in the mice, and their behavior appeared normal, with no signs of allergic reactions. Subsequent autopsy examinations and histopathological slice analyses also did not reveal any tumor tissues or signs of lesions. Further analysis shows that although the EOS# value in the MG-MT 30% group was significantly higher than the control group, these values still fall within the normal physiological range. Additionally, the P-value only reflects the statistical significance of differences between data; while the magnitude of differences can be derived from the original data themselves, the P-value provides no information regarding the practical implications of these differences. Based on the above evidence — no associated adverse symptoms in clinical observations, no pathological lesions in autopsies, EOS# values remaining within the normal physiological range, and P-values not indicating practical biological significance — we tend to believe that the increase in EOS# is not directly caused by feeding MSTN gene-edited Mongolian beef cattle. To ensure the accuracy and scientific validity of hematological parameter analysis, it is also necessary to comprehensively consider multiple factors, including individual animal differences, experimental conditions, and potential non-specific responses.

Compared to traditional serological tests, serum metabolomics can accurately detect and analyze a wide range of metabolites commonly found in serum. These metabolites can comprehensively and effectively reflect and assess the metabolic status of mice. We investigated whether there were significant differences in serum metabolites across different feeding groups of mice^25–27^. In this study, metabolomics analysis did not cover all feeding groups; instead, we focused on three key groups for comparison: the control group (CON), 30% wild-type Mongolian beef feeding group (MG-WT 30%), and 30% MSTN gene-edited Mongolian beef feeding group (MG-MT 30%). This targeted strategy was based on the “high-dose priority principle” in toxicological research: high-dose groups represent the maximum exposure of mice to the test substance (beef), making them more likely to show potential metabolic abnormalities or deviations from the normal state than low-dose groups. If no significant metabolic disorders or obvious differences from the control group are observed in these high-dose groups, it can be reasonably inferred that lower-dose groups (e.g., 10% and 20% feeding groups) will not induce more prominent metabolic changes. This design not only ensures the scientific validity of evaluating the metabolic safety of MSTN gene-edited Mongolian beef but also avoids redundant analysis of low-dose groups (which rarely provide additional meaningful information), optimizing data interpretation efficiency while maintaining research rigor. As observed in this study, the degree of separation of PCA (Principal Component Analysis) for metabolites in each group of mice was not significant. QC samples clustered together, and there were notable interactions among the groups, further confirming the reliability of the data. This suggests that the type of feed (whether wild-type Mongolian beef or genetically edited Mongolian beef) does not have a significant impact on the overall metabolic status of the mice.

We further analyzed the differences in the types and quantities of metabolites between the female and male mouse control groups and the MG-WT 30% group, as well as the MG-MT 30% experimental group. We found significant differences (*p* < 0.01), with fewer metabolites and similar metabolic pathways, which reaffirmed the similarity in metabolite content and types across the groups, as well as the limited impact of feed type on the metabolic state of the mice. Specifically, when we compared the CON vs. WT 30% group as a background with the MG-MT 30% group, we were able to identify the differential metabolites produced in this context. This finding suggests these differential metabolites are more likely related to beef addition, rather than whether the beef is wild-type or gene-edited. This conclusion about the generation of these differential metabolites offers a new perspective on understanding the impact of beef supplementation on the metabolic state of mice. It is important to note that while serum metabolomics was a key technical approach in this study—consistent with its recognition as one of the most innovative tools for comprehensively understanding biological safety—our current interpretation of inter-group differential metabolites remains relatively brief. This was a deliberate design choice aligned with the core objective of this study: we prioritized verifying the “overall metabolic safety” of MSTN gene-edited Mongolian beef, focusing first on confirming whether high-dose feeding would disrupt mouse metabolic homeostasis (as reflected by PCA clustering and limited metabolite differences). Thus, in-depth work such as the identification of key metabolites (especially those potentially persistently affected by MSTN gene-edited beef), pathway annotation, and detailed biological interpretation was not fully expanded in this study. We recognize that such in-depth analysis is critical for enhancing the translational value of the research, and therefore plan to address these aspects in subsequent studies, including integrating multi-omics data to clarify the regulatory mechanisms of differential metabolites and extending the experimental cycle to verify persistent metabolic effects.

The scope of serum metabolomics testing includes all metabolites in the serum. The range of detectable metabolites is broad, and the physiological metabolic changes in mice are interconnected and influenced by various external factors, such as the mice’s specific responses or inflammation^26^, the use of different anesthetics^28^, and the separation gel components in serum collection tubes and anticoagulants, which can affect metabolite concentrations or generate some atypical metabolites^29^. To minimize these confounding effects, our study strictly controlled experimental conditions: all mice were housed under standardized environmental conditions with ad libitum access to food and water; the same type of anesthetic was used for euthanasia; serum was collected using tubes without separation gel, and EDTA was used as the anticoagulant consistently across all groups. Additionally, each experimental group included 6 biological replicates to ensure data reproducibility. Furthermore, although standardized mouse experiments significantly reduce inter-individual variability, lifestyle, and dietary influences, different experimental protocols, mouse strains, and sampling locations could still have some impact on the experimental results^30^. Nevertheless, the unique value of metabolomics analysis in revealing metabolic changes in complex biological processes is irreplaceable. We not only obtained comprehensive information about the metabolic status of the mice but also explored the impact of feed types on their metabolic state, providing valuable insights for future research.

At the end of the 90-day subchronic toxicity test, detailed gross dissections of the chest and abdomen of KM mice were carried out strictly following standard procedures. Observations were made carefully, recording features such as organ size, color, and texture. No pathological changes were found in the organs of any group of mice. To further confirm, key organs of the mice, including the heart, liver, spleen, lungs, left kidney, right kidney, testes, and ovaries, were removed for weighing. By comparing the relative organ coefficients between the control and treatment groups, no statistically significant differences were observed, which further supported our initial findings. For more in-depth pathological information, we selected the heart, liver, spleen, lungs, kidneys, and gonads (including testes and ovaries) from each group of mice for histopathological examination, allowing for a more intuitive understanding of the tissue’s cellular structure and arrangement. No signs of pathological changes were observed in these tissues, indicating that MSTN gene-edited Mongolian beef had no impact on the circulatory, blood, immune, respiratory, or urinary systems of KM mice.

To the best of our knowledge, there have been no reports on the safety evaluation of MSTN gene-edited Mongolian beef in mice until now. Our study results show that MSTN gene-edited Mongolian beef demonstrated good safety in subchronic toxicity tests, with no significant negative effects on the main physiological systems of KM mice. Additionally, adding the MSTN gene-edited Mongolian beef appeared to yield similar results to those of wild-type Mongolian beef in various tests. Furthermore, the results of this study are consistent with those of previously reported feeding studies on MSTN and FGF5 double-knockout sheep meat in Wistar rats, as well as the feeding of MSTN gene-edited pork for 90 days in rats^31,32^. Study on feeding with MSTN and FGF5 double-knockout lamb meat: Rats showed no toxic reactions, and there were no significant abnormalities in growth and organ indicators; the safety of double-knockout lamb meat was equivalent to that of wild-type lamb meat. Study on feeding with MSTN gene-edited pork: In the 90-day high- and low-dose experiment, rats had no subchronic toxicity, with normal liver and kidney function as well as blood and immune indicators; moreover, the edited pork had lower fat content and no exogenous proteins. These two studies are consistent with this study, all demonstrating that MSTN-related gene-edited meats are non-toxic, do not damage organs, and have both safety and meat quality optimization value.

The core advantages of this study are primarily reflected in: the separate analysis of male and female mouse data to examine the impact of gender differences on the experimental results; reference to various relevant experimental standards and recommendations, with the addition of MSTN gene-edited Mongolian beef at multiple varying ratios into the mice feed, ensuring the rigor of the experimental design and the reliability of the results. Furthermore, we have discarded traditional serological testing methods in favor of more advanced and advantageous serum metabolomics analysis, allowing for more precise detection of potential biomarker changes. However, it must be acknowledged that this study does have certain limitations, with a relatively limited sample size. To more comprehensively and deeply assess the safety of MSTN gene-edited Mongolian beef, we have planned a series of follow-up studies, including but not limited to acute toxicity tests, chronic toxicity studies lasting 12 months or longer, and specialized analyses of potential allergenicity and nutritional properties of MSTN gene-edited Mongolian beef. These subsequent studies will not only further supplement the conclusion regarding the safety of MSTN gene-edited Mongolian beef but also lay a more solid scientific foundation for the safe and rational application of MSTN gene-edited Mongolian beef in human diets.

## Conclusion

Our research results show that no signs of toxicity or adverse reactions were observed in any of the mouse groups involved in the trial. Given that this trial encompasses multiple factors such as environment, age, and genetic background, we confirm that within the framework of this study, all research groups were consistent in these factors, except for the type of diet. Statistical analysis revealed no significant differences between the groups in terms of weekly weight gain and total food consumption. Compared to the commercial control group, while some statistically significant differences were observed in hematological parameters, serum metabolomics analysis, and relative organ weights (i.e., the ratio of organ weight to body weight) between the three groups of mice fed with MSTN gene-edited Mongolian beef, these differences were all within the normal physiological range. Therefore, we believe these differences may stem from natural individual variations between mice rather than being directly caused by the dietary factors. Furthermore, histopathological examination of the organs did not reveal any diet-related abnormal changes. The overall analysis suggests that the observed differences are not directly linked to the long-term consumption of MSTN gene-edited Mongolian beef in the feed. Based on this, we conclude that in this 90-day subchronic toxicity study, MSTN gene-edited Mongolian beef did not exhibit any toxic effects on KM mice.

## Materials and methods

### Animal diet preparation

According to our preliminary research, using beef derived from wild Mongolian Cattle and beef derived from MSTN gene-edited Mongolian Cattle as experimental materials, which were provided by the experimental base of Inner Mongolia University. The beef was ground into a paste and added to the feed to partially replace the protein components of commercial feed. The feed was prepared by SPF (Beijing) Biotechnology Co., Ltd. and divided into seven groups: Con: the standard commercial feed for mice was used as the control group; MG - WT10%: the standard commercial feed for mice with beef derived from wild Mongolian Cattle added to replace 10% of the protein; MG - WT20%: the standard commercial feed for mice with beef derived from wild Mongolian Cattle added to replace 20% of the protein; MG - WT30%: the standard commercial feed for mice with beef derived from wild Mongolian Cattle added to replace 30% of the protein; MG - MT10%: the standard commercial feed for mice with beef derived from MSTN-knockout Mongolian Cattle added to replace 10% of the protein; MG - MT20%: the standard commercial feed for mice with beef derived from MSTN-knockout Mongolian Cattle added to replace 20% of the protein; MG - MT30%: the standard commercial feed for mice with beef derived from MSTN-knockout Mongolian Cattle added to replace 30% of the protein (Table 5)^33^. After sterilization with 60Co radiation, the ration met the criteria for a clean rat feeding ration.

### Experimental animals

A total of 120 KM mice, 4 weeks old and weighing 23–27 g with an equal ratio of males to females, were acquired from SPF (Beijing) Biotechnology Co., Ltd. (Laboratory Animal Production License No. SCXK (Beijing) 2024-0001). Upon arrival, the KM mice were acclimatized in the animal laboratory for one week, during which daily clinical observations and records were conducted. At the conclusion of the adaptation period, any animals exhibiting abnormalities or with body weights outside the specified range were excluded before proceeding with subsequent tests. The experiment commenced when the KM mice reached 5 weeks of age, and the animals were randomly housed in stainless steel wire cages. The rationale for the inconsistent number of animals in each group in the experimental design is that KM mice have a small blood volume; to ensure sufficient quantities of blood and serum are obtained during collection, 6 additional mice were included in each of the three concentration groups for serum analysis, and the number of animals in each group meets the statistical requirements for subsequent data analysis.

### Feeding environment and management

The mice were raised in the SPF Laboratory of the Laboratory Animal Research Center, Inner Mongolia University. Before the experiment, the whole mouse room was closed and disinfected with disinfectant and ultraviolet light. The indoor shelves, other facilities, and feeding and drinking equipment were cleaned and disinfected. The mice were housed in an environmentally controlled room with the temperature at 20–24 ℃, 40–70% relative humidity, a 12 h artificial light/dark cycle, and 15 air changes/h. The indoor sanitation was cleaned every day, the air was kept clean, and the beds were replaced regularly. The feeding lasted for 90 days.

### Clinical observation

We referred to the recommendations proposed by the European Food Safety Authority (EFSA) in 2014 regarding 90-day feeding studies and formulated the animal health and welfare standards for 90-day feeding studies^34^. During the 90 days of feeding, the appearance and behavior of the mice were observed twice a day, and indicators such as hair color, limbs, eyes, nerve activity, breath, tail, and number of secretions were compared between the different groups.

### Body weight and mean weekly feed utilization

Record the mice’s body weight, total food intake, and body weight gain, and other growth performance indicators weekly. Calculate the average weekly feed utilization rate (%) = (weekly single-cage weight gain/weekly single-cage feed consumption) × 100%^35^.

### Routine blood tests

At the end of the experimental period, the mice were fasted for 12 h, then anesthetized, and blood samples were collected through ocular bleeding. Anticoagulated blood was harvested for clinical testing. Hematological parameters were assessed using the Pentra XL80(ABX, France)automated hematology analyzer^36^, including White Blood Cell count (WBC), Lymphocyte count (LYM#), Lymphocyte percentage (LYM%), Monocyte count (MON#), Monocyte percentage (MON%), Eosinophil count (EOS#) Eosinophil percentage (EOS%), Basophil count (BAS#), Basophil percentage (BAS%), Neutrophil count (NEU#), Neutrophil percentage (NEU%), Red Blood Cell count (RBC), Hematocrit (HCT), Mean Corpuscular Volume (MCV), Red Cell Distribution Width (RDW), Mean Corpuscular Hemoglobin (MCH), Mean Corpuscular Hemoglobin Concentration (MCHC), Platelet count (PLT), Plateletcrit (PCT), Platelet Distribution Width (PDW), and Mean Platelet Volume (MPV).

### Non-targeted serum metabolomics analysis

We collected the blood samples from mice in the control group (Con), MG-WT30%, and MG-MT30%, and placed them into tubes without anticoagulant. Centrifuged these samples at 3000 rpm for 15 min, and then obtained the serum. The gathered samples were thawed over ice, with metabolites extracted using an 80% methanol buffer^37^. Additionally, pooled QC samples were prepared through the combination of 10 µL aliquots from each extraction mixture. Raw data underwent conversion via MSConvert, followed by peak extraction utilizing XCMS^38^. Adduct and ion annotation were carried out with CAMERA, while final identification was accomplished using metaX, incorporating a library derived from an in-house standard compound database^39^. For metabolite annotation, online KEGG and HMDB databases were employed, involving the matching of precise molecular mass data (m/z) from samples against those within the databases^40^. After data processing and annotation, log₂ transformation was used for data normalization, and group mean imputation for missing values (missing rate ≤ 20%) while excluding metabolites with missing rate >20% for data quality control; principal component analysis (PCA) was performed to assess sample repeatability and inter-group separation; two-tailed independent samples t-test was used to verify the statistical significance of metabolite abundance differences between groups; combined with fold change (FC) and variable importance for projection (VIP) values, significant differential metabolites were screened, and volcano plots and Venn diagrams were drawn to visualize differential metabolite distribution and overlap.

### Organ weight and histopathological analysis

Euthanize the mice and dissect out the organs, including the heart, liver, kidney, spleen, lungs, testes, and ovaries. Weigh these organs and observe the gross appearance of the major organs, as well as the abdominal and thoracic cavities. Subsequently, samples from these organs were taken for histopathological evaluation, encompassing the heart, liver, spleen, lungs, kidney, ovaries, and testes. The relative organ weight (ROW) of each animal was calculated using the formula: Relative Organ Weight (%) = (Organ Weight/Body Weight) × 100^41^. The collected organs were immersed in 4% neutral formalin for fixation. These samples were processed into paraffin-embedded tissue sections and stained with hematoxylin and eosin (HE) using routine methods^42^. Histopathological analysis was conducted under a microscope to observe differences between the experimental and control groups.

### Statistical analysis

SPSS 27.0 statistical software (IBM Corporation, Somers, NY, USA) was used for statistical analysis. All values are presented as mean ± SD. One-way ANOVA was used to analyze the significance of pairwise differences, and Tukey’s multiple comparison test was used to analyze the significance of pairwise differences. *P* < 0.05 was considered statistically significant.

## Supplementary Information

Below is the link to the electronic supplementary material.

Supplementary Material 1

## Data Availability

The data that support the findings of this study are available from the first author upon reasonable request.
