# Supplementary material for: A 90-day safety study of meat from MSTN gene-edited Mongolian cattle in mice
Source: Sci Rep. 2026 Jan 15;16:2091. doi: 10.1038/s41598-025-31934-x (PMC12808327; doi:10.1038/s41598-025-31934-x)
Supplement: Supplementary file 1 — Supplementary Material 1 [file 41598_2025_31934_MOESM1_ESM.docx]

| \|  \| **Heart** \| **Liver** \| **Spleen** \| **Lung** \| **Kidney** \| **Testis** \| \| --- \| --- \| --- \| --- \| --- \| --- \| --- \| \| **MG-WT 10％** \| 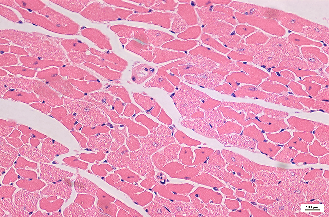 \| 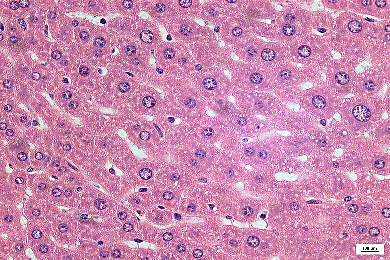 \| 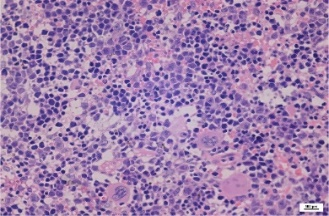 \| 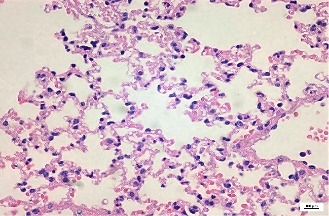 \| 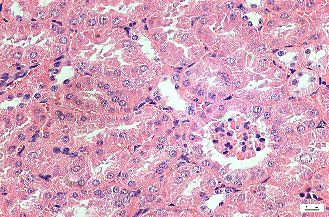 \| 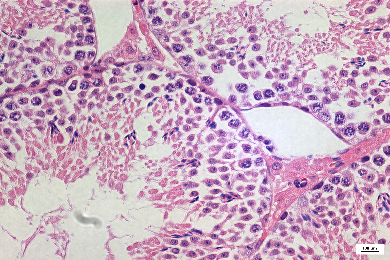 \| \| **MG-WT 20％** \| 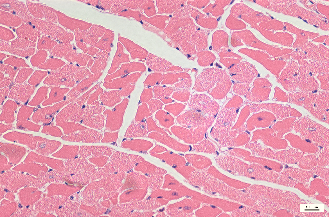 \| 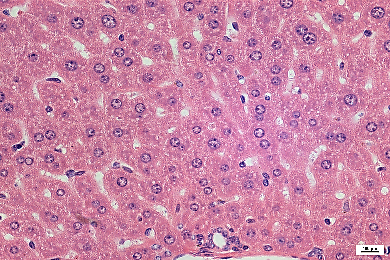 \| 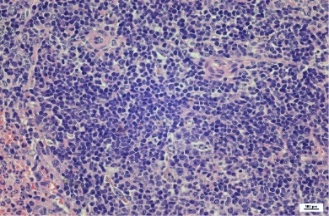 \| 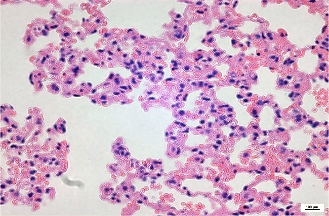 \| 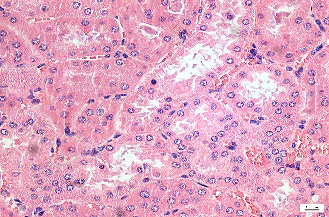 \| 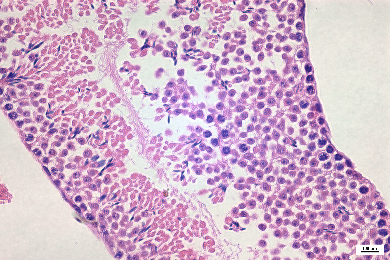 \| \| **MG-MT 10％** \| 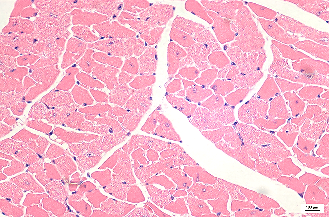 \| 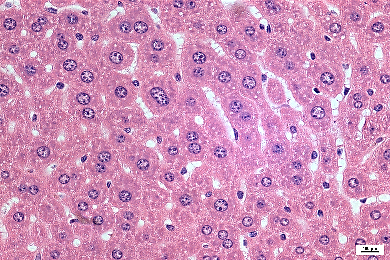 \| 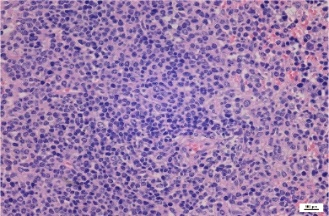 \| 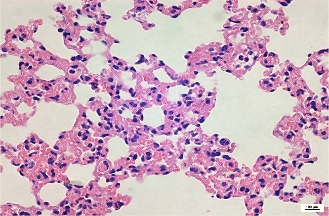 \| 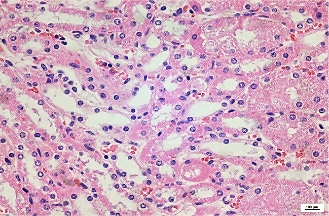 \| 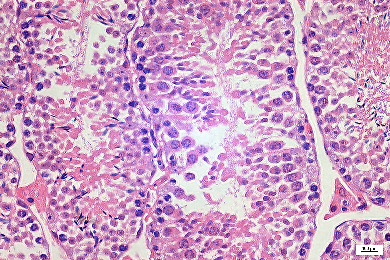 \| \| **MG-MT 20％** \| 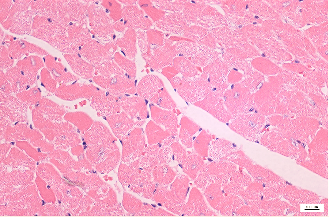 \| 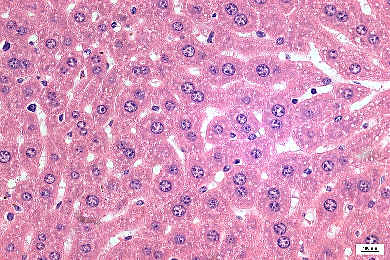 \| 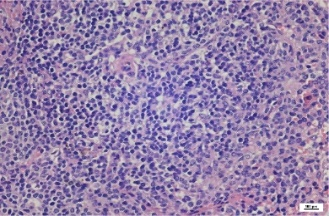 \| 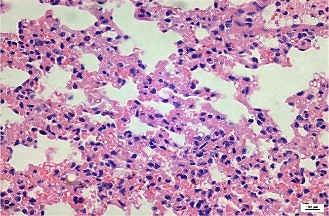 \| 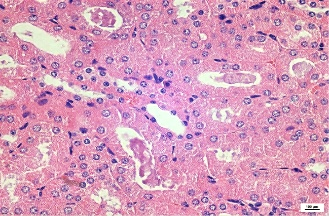 \| 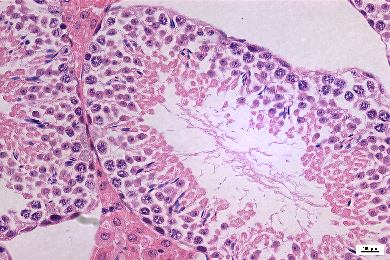 \| |
| --- | --- | --- | --- | --- | --- | --- | --- | --- | --- | --- | --- | --- | --- | --- | --- | --- | --- | --- | --- | --- | --- | --- | --- | --- | --- | --- | --- | --- | --- | --- | --- | --- | --- | --- | --- |

Fig.S1 Tissue histopathology at 90 day, including heart、liver、spleen、lung、kidney and testis. MG - WT10%: the standard commercial feed for mice with beef derived from wild Mongolian Cattle added to replace 10% of the protein; MG - WT20%: the standard commercial feed for mice with beef derived from wild Mongolian Cattle added to replace 20% of the protein; MG - MT10%: the standard commercial feed for mice with beef derived from MSTN-knockout Mongolian Cattle added to replace 10% of the protein; MG - MT20%: the standard commercial feed for mice with beef derived from MSTN-knockout Mongolian Cattle added to replace 20% of the protein. Tissues of rats stained with H&E(40X).

|  | **Heart** | **Liver** | **Spleen** | **Lung** | **Kidney** | **Ovary** |
| --- | --- | --- | --- | --- | --- | --- |
| **MG-WT 10％** | 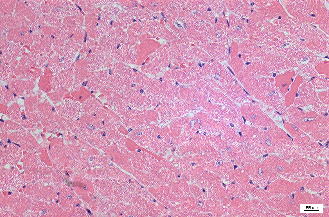 | 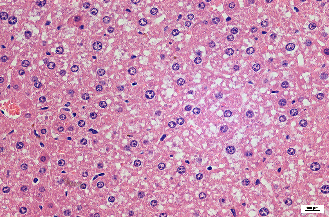 | 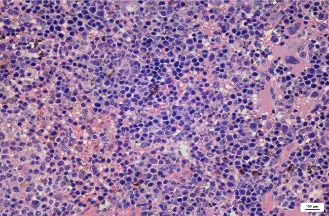 | 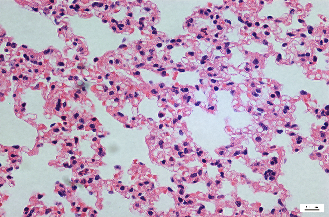 | 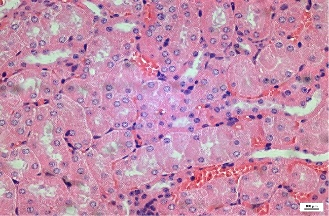 | 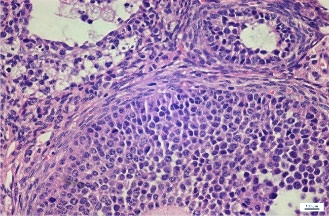 |
| **MG-WT 20％** | 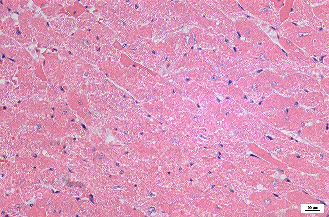 | 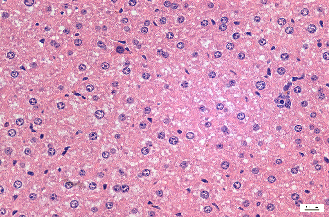 | 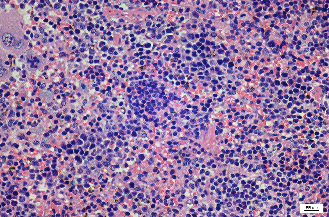 | 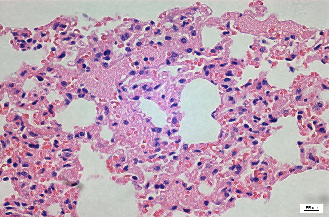 | 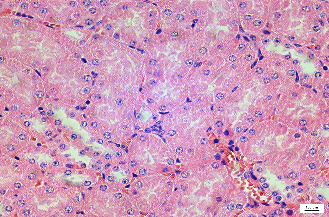 | 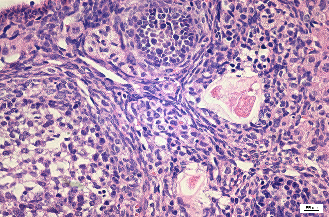 |
| **MG-MT 10％** | 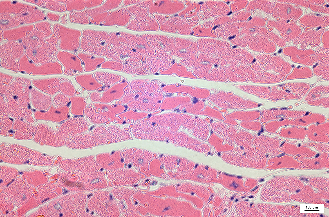 | 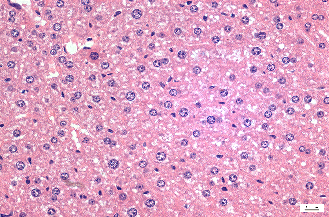 | 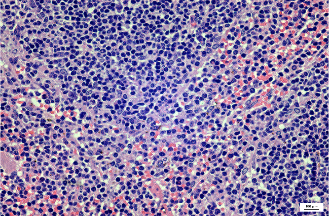 | 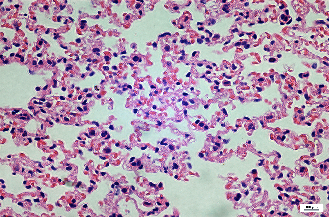 | 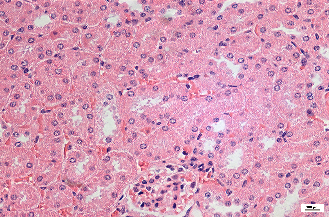 | 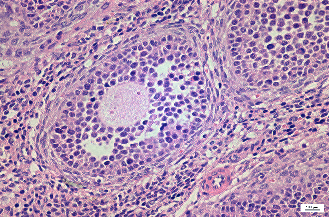 |
| **MG-MT 20％** | 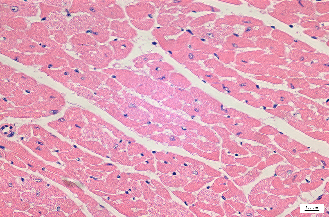 | 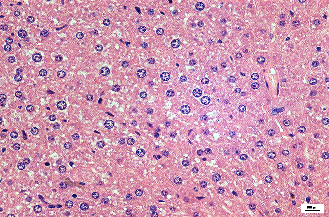 | 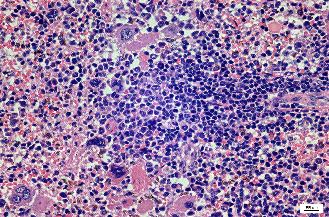 | 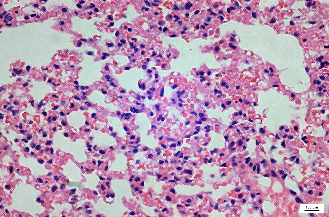 | 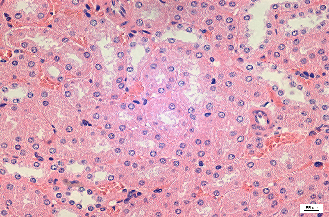 | 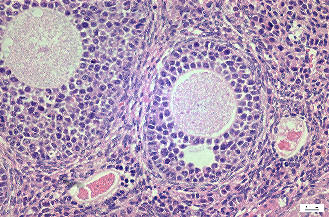 |

Fig.S2 Tissue histopathology at 90 day, including heart、liver、spleen、lung、kidney and ovary. MG - WT10%: the standard commercial feed for mice with beef derived from wild Mongolian Cattle added to replace 10% of the protein; MG - WT20%: the standard commercial feed for mice with beef derived from wild Mongolian Cattle added to replace 20% of the protein; MG - MT10%: the standard commercial feed for mice with beef derived from MSTN-knockout Mongolian Cattle added to replace 10% of the protein; MG - MT20%: the standard commercial feed for mice with beef derived from MSTN-knockout Mongolian Cattle added to replace 20% of the protein. Tissues of rats stained with H&E(40X).
